# Effect of Process Conditions on the Properties of Resorcinol-Formaldehyde Aerogel Microparticles Produced via Emulsion-Gelation Method

**DOI:** 10.3390/polym13152409

**Published:** 2021-07-22

**Authors:** Seeni Meera Kamal Mohamed, Charlotte Heinrich, Barbara Milow

**Affiliations:** Department of Aerogels and Aerogel Composites, Institute of Materials Research, German Aerospace Center (DLR), Linder Hoehe, 51147 Cologne, Germany; charlotte.heinrich@dlr.de (C.H.); barbara.milow@dlr.de (B.M.)

**Keywords:** aerogel, resorcinol-formaldehyde, emulsion-gelation, microparticles, supercritical drying

## Abstract

Organic aerogels in the form of powder, microgranules and microsized particles receive considerable attention due to their easy fabrication, low process time and costs compared to their monolithic form. Here, we developed resorcinol-formaldehyde (RF) aerogel microparticles by using an emulsion-gelation method. The main objective of this study is to investigate the influence of curing time, stirring rate, RF sol:oil ratio and initial pH of the sol in order to control the size and properties of the microparticles produced. The emulsion-gelation of RF sol prepared with sodium carbonate catalyst in an oil phase at 60 °C was explored. RF microparticles were washed with ethanol to remove the oil phase followed by supercritical and ambient pressure drying. The properties of the dried RF microparticles were analyzed using FT-IR, N_2_ adsorption isotherm, gas pycnometry, wide angle X-ray scattering and scanning electron microscope. RF microparticles with high surface area up to 543 m^2^/g and large pore volume of 1.75 cm^3^/g with particle sizes ranging from 50–425 µm were obtained.

## 1. Introduction

Aerogels are ultra-lightweight materials with a highly open porous nature with more than 90% *v*/*v* of empty space. They are defined as interconnected solid colloidal or polymeric nanoparticles expanded throughout their volume by gas [1]. This contributes to their interesting properties such as high surface area, ultra-low thermal conductivity, low density, high porosity, etc. [2]. These properties make aerogels promising materials for various applications such as thermal and acoustic insulation, catalysis, gas storage, energy storage, pollutant removal, sensors, space dust collector, etc. [3,4,5].

Resorcinol-formaldehyde (RF) aerogel is an important class of organic aerogels with the above properties which largely depend on the synthesizing and processing conditions [6]. They are the major source for the preparation of carbon aerogels with remarkable electrical, thermal and mechanical properties [7]. Carbon aerogels find applications in electrodes, batteries, foundry industries, adsorbents, catalysis, etc. [8,9,10]. In particular, the foundry industry can take considerable advantage of, e.g., energy efficiency by using open porous nanostructured materials like aerogels as binders and additives for sand cores and molds [9,11]. In the past, organic and inorganic aerogels in the form of granules were used as an additive to improve the properties of sand cores and casted parts such as metal penetration, surface fineness and collapsibility [12]. Additionally, high temperature stable aerogels present in the sand cores could be able to absorb gases evolved during the decomposition of polymeric resins employed as sand grain binders [13].

Brück and Ratke [9] utilized RF-based organic aerogels for the first time as a new binding material for sand core and mold production. A new kind of sand called AeroSand, a composite of conventional sand and RF aerogel, was developed by Ratke and Brück [14]. The aerogel-bonded sand provided the advantage of easy core removal without any mechanical force. Moreover, pyrolysis was applied to convert RF aerogels into carbon aerogels and further RF AeroSand into Carbon AeroSand [15]. The transformation of RF aerogels into amorphous carbon aerogels allows them to bind firmly with sands and also act as additives. Further, recycling of Carbon AeroSand was achieved by heating at 400 °C without formation of any organic waste or volatile byproducts such as benzene, toluene and xylene (BTX). However, Carbon AeroSands have poor stability compared to RF AeroSands. Hence, drying and curing of RF AeroSand was recommended at lower temperatures under vacuum instead of direct pyrolysis to avoid reduction in stability. Meyer et al. [13] utilized silica-based aerogels in granular form as an additive and examined the sand core behavior and cast surface quality. They observed that aerogel additives exhibited high thermal stability compared to conventional additives and also enhanced the gas permeability. Generally, aerogels are pulverized to match the size of the sand grain in order to be used as nanoadditives for the sand–binder system in foundries [12]. Hence, industrial scale use at the foundries mainly requires aerogel granulate materials, not bulk monoliths, as binder and additive. For many years, silica aerogels have been used in casting operations as molds [16]. Considering the above, RF aerogels and their carbon counterpart require much attention from the industry point of view to solve the problems associated with cores and molds [12].

The traditional method of RF aerogel monolith preparation comprises aqueous polycondensation of resorcinol with formaldehyde under alkaline condition using sodium carbonate as catalyst [2]. However, milling and grinding of thus obtained monoliths to specific size is not an easy task. Thus, grinded particles have broad particle size distribution, are irregularly shaped and exhibit poor hydraulic performance [17]. Hence, an alternative approach is required to produce RF aerogel granules. The synthesis in the form of microparticles is of interest because of their easy fabrication, reduced process time, equipment costs and easy handling [18]. There are various methods available to prepare aerogel microparticles, such as dropping, jet-cutting, prilling by vibration, spray drying, emulsion-gelation, etc. [19].

Emulsion-gelation method is an important technique that helps to produce larger scale aerogel microparticles in a robust and controlled way [20,21]. In this method, RF sol as aqueous phase is added to continuous oil phase while constantly being stirred to form emulsion microdroplets. The microdroplets will undergo cross-linking/gelation under the influence of heating to form stable cross-linked microspheres. These microspheres will be separated by sedimentation and washed, followed by solvent exchange and supercritical drying to obtain aerogel microparticles [22]. Mayer et al. [23] first reported the preparation of RF aerogel microspheres using an inverse emulsion polymerization method. There are a number of findings available in the literature on the preparation of RF microspheres and their carbon counterpart [24,25,26,27,28]. On the contrary, detailed physical and chemical characteristics of the RF aerogel microparticles were not studied in comparison with their monoliths and carbonized products [1].

In the present work, we report the preparation of RF aerogel microparticles by using an emulsion-gelation method. Emulsion-gelation method offered us an easy route to produce different microparticles by varying the process conditions. In addition, the detailed investigations of the microparticles were analyzed using FT-IR spectroscopy, N_2_ adsorption isotherm, gas pycnometry, wide angle X-ray scattering and scanning electron microscopy.

## 2. Materials and Methods

### 2.1. Materials

Resorcinol (98%) and sodium carbonate (anhydrous, 99.8%) were purchased from Sigma Aldrich, Schnelldorf, Germany. Formaldehyde (23.5%), low-methanol solution was procured from Carl Roth, Karlsruhe, Germany. Rapeseed oil was obtained from a local market. Ethanol (99.9%) was obtained from Th. Geyer, Renningen, Germany. Deionized water was used throughout the experiments. KBr for IR spectroscopy was obtained from Merck, Darmstadt, Germany. All the reagents were used as received without any further purification.

### 2.2. Preparation of RF Aerogel Microparticles

**Scheme 1:**
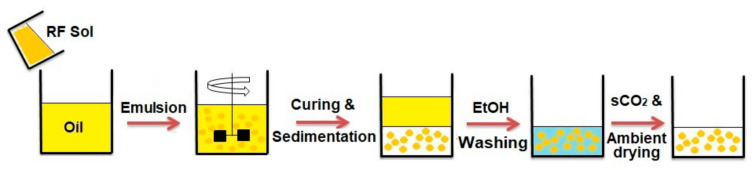
Preparation of RF aerogel microparticles.

The RF aerogel microparticles were prepared by using a modified emulsion-gelation method as follows [29]: first, aqueous RF sol phase was prepared by mixing appropriate quantities of resorcinol (R), formaldehyde (F), water (W) and sodium carbonate (C) at room temperature under continuous stirring at a rate of 250 rpm. The molar ratios of R/C, R/F and R/W were maintained as 200, 0.74 and 0.044, respectively. After mixing all the reactants, the stirring was continued for another 30 min to obtain the desired RF sol, and initial pH of the sol was ~7. Then, RF sol was added slowly into the oil phase containing rapeseed oil under stirring of 200 rpm. The oil phase was preheated at 60 °C in a water bath. The interfacial tension between RF sol and rapeseed oil phases was found to be 4.57 ± 0.09 mN/m. The stirring was continued for another 3 h until cross-linked RF spheres formed. Then, the suspension containing oil and RF spheres was kept in an oven at the same temperature for further curing and aging for different time intervals, viz., 14, 24, 48 and 72 h. Later, the spheres were settled gravimetrically and the top oil layer was separated by decanting. Further, spheres were washed several times with ethanol in order to remove the remaining oil and also to exchange the water present in the spheres. The water content in the ethanol was frequently checked until it reached below 5%. The alcogel spheres were further dried using supercritical CO_2_ drying (SCD) and ambient pressure drying (APD) methods. The schematic diagram of RF aerogel microparticle preparation is depicted in Scheme 1 and the pictures of the microparticles are given in Figure S1 (Supplementary Material). RF microparticles have skeletal densities in the range of 1.22–1.42 g/cm^3^ reported for RF monoliths [30]. The sample details and experimental conditions are presented in Table 1.

### 2.3. Characterization

The ambient pressure drying of the microparticles was carried out in an oven (Memmert Universal Oven UF110, Memmert GmbH + Co. KG, Schwabach, Germany) at 60 °C. The microparticles were also dried using the supercritical CO_2_ method in an autoclave (Eurotechnica GmbH, Bargteheide, Germany) at 115 bar and 60 °C after washing with ethanol several times. The skeletal densities (ρ_s_) of the microparticles were obtained using AccuPyc II 1340 Helium Pycnometer from Micromeritics, Unterschleissheim, Germany. The interfacial tension between the dispersed (RF sol) and continuous (rapeseed oil) phases was performed using Du Noüy ring method (Krüss Force Tensiometer-K100C, KRÜSS GmbH, Hamburg, Germany). The surface area, pore volume and pore size distribution of the microparticles were measured using N_2_ adsorption/desorption isotherm analysis using MicroActive 3Flex 3500 Gas Sorption Analyzer from Micromeritics, Unterschleissheim, Germany, at 77.3 K temperature with a relative pressure (P/P_0_) range of 0.01–1.0. The samples were pretreated with Smart VacPrep 067 HIVAC (Micromeritics, Unterschleissheim, Germany) at 120 °C for 12 h before starting the measurements. The surface areas (S_BET_) were calculated using Brunauer–Emmett–Teller (BET) method and had positive C value. Pore size distribution (PSD) curves were obtained using the Barrett–Joyner–Halenda (BJH) method. The total pore volume (V_p_) was estimated from the adsorption branches of the isotherm at relative pressure (P/P_0_) of 0.991. The pore sizes (D_p_) were obtained from the maxima in the BJH pore size distribution curves. Infrared spectra of the RF microparticles were recorded using Fourier transform infrared (FT-IR) spectrometer, Bruker-Tensor 27 instrument (Bruker, Ettlingen, Germany) with a resolution of 4 cm^−1^ and 100 scans over the range of 4000–400 cm^−1^ by making them as pellets using KBr. Wide angle X-ray scattering (WAXS) measurements were attained using Bruker D8 Advance (Bruker, Karlsruhe, Germany) with Cu Kα radiation (λ = 1.5406 Å) at 35 kV and 30 mA and the goniometer had a radius of 280 mm. The detector is a LynxeExe XET from Bruker, Karlsruhe, Germany. The conditions for the measurements were: (i) scanning ranges from 10–100° of 2θ with a step size of 0.01° and the time/step was 3 s; (ii) primary slit: fixed divergence slit with 0.22° to illuminate the sample, secondary slit: 5.232 mm (fully opened), detector opening: 2.305°. The mathematical detector slit was 0.012° for the resolution and the primary and secondary Soller-slit was 2.5°. The samples were dry grinded to a suitable size with a mortar and pestle. The powder was pressed manually in a small cavity in a single crystal with 5 mm diameter. The single crystal was a low-background-holder for small amounts of powder and provided no peaks in the measured 2θ range. The size and morphology of the RF microparticles were viewed using a ZEISS ULTRA55 scanning electron (SEM) (Zeiss SMT, Oberkochen, Germany) with a low operating voltage of 2–3 kV. All the samples were sputtered using a thin layer of platinum using BAL-TEC SCD 500 Sputter Coater (BALTIC, Wetter/Ruhr, Germany) with a current of 21 mA for 60 s. The sizes of the microparticles were measured using open source ImageJ software.

## 3. Results and Discussion

### 3.1. FT-IR Analysis

**Figure 1:**
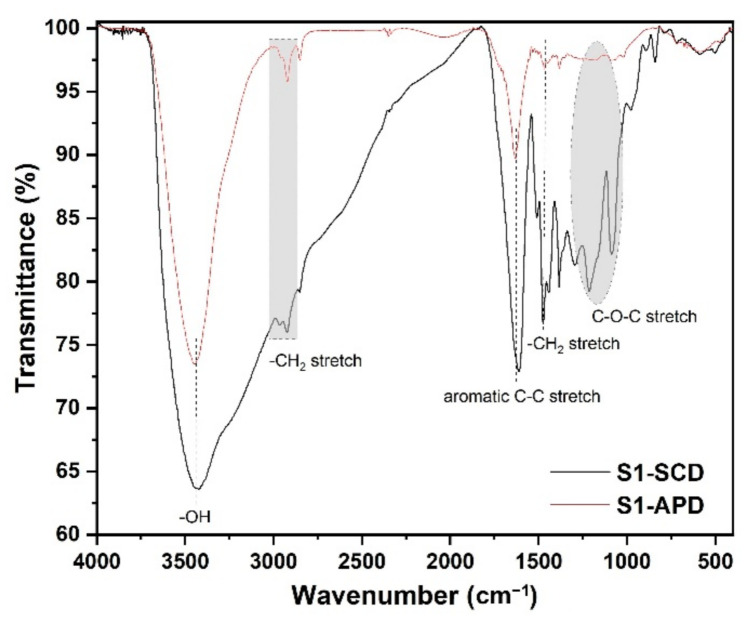
FT-IR spectra of RF aerogel microparticles.

Figure 1 shows the FT-IR spectra of the RF aerogel microparticles dried by SCD and APD. IR spectra of microparticles show the same features of the sodium carbonate catalyzed monolithic aerogel reported in the literature [31]. A broad peak is observed at around 3440 cm^−1^ due to -OH groups present in the benzene ring of resorcinol and also due to -CH_2_-OH groups attached to resorcinol molecule, which do not involve the polycondensed network formation. The peaks at 2920 and 1470 cm^−1^ correspond to -CH_2_ stretching vibration and another peak at around 1610 cm^−1^ represents C-C stretching in the aromatic ring. The characteristic peaks at 1220 and 1090 cm^−1^ can be assigned to stretching vibration of C-O-C linkage of the methylene ether derivative formed by the polycondensation of hydroxymethyl resorcinol resulting from the reaction between resorcinol and formaldehyde. There are no significant changes observed in the IR characteristic absorption of the microparticles dried by two different methods except broadness and intensity of the peaks in the case of supercritical dried microparticles.

### 3.2. WAXS Analysis

**Figure 2:**
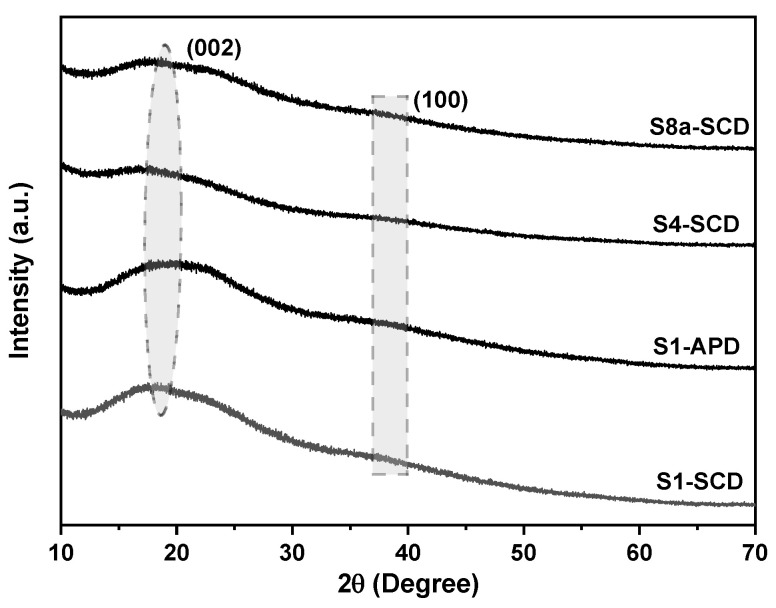
WAXS pattern of RF aerogel microparticles.

The WAXS pattern of the RF microparticles prepared with different conditions is presented in Figure 2. A broad beak ranging from 10 to 30° represents the amorphous nature of the RF microparticles. The broader peak centered approximately at around 20° corresponds to characteristic 002 diffraction. Further, a non-prominent peak is observed at around 40° corresponding to characteristic 100 diffraction. These two peaks are assigned to amorphous carbon in the RF microparticles [32]. Such a kind of XRD pattern with dominant 002 and unnoticed 100 diffraction peaks for pristine RF resin was reported by Kumar and Kandasubramanian [33]. All the RF microparticles exhibit a similar kind of XRD pattern irrespective of the preparation conditions.

### 3.3. N_2_ Adsorption/Desorption Isotherm

**Figure 3:**
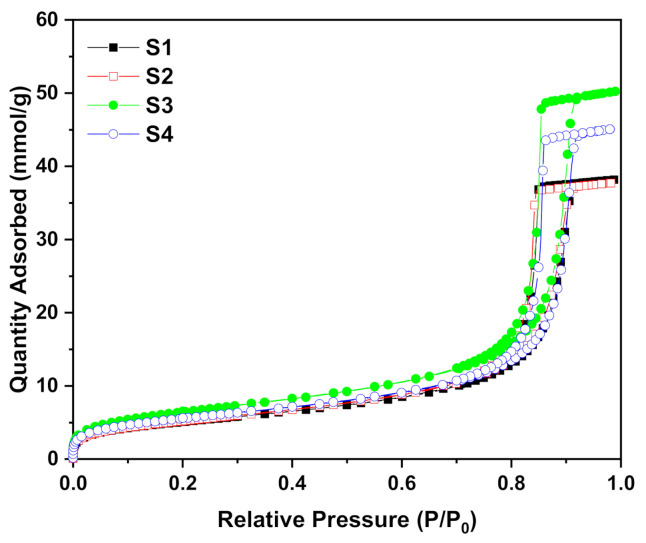
Nitrogen physisorption isotherms (77K) for the prepared RF microparticles dried by SCD.

The typical adsorption/desorption isotherms of the RF microparticles are presented in Figure 3. All the supercritical dried RF microparticles exhibit Type IV(a) isotherm with H2 hysteresis loop according to 2015 IUPAC classification [34]. The presence of H2 loop at relative pressure (P/P_0_) range (0.75–0.92) indicates that the microparticles possess mesopores. This type is associated with the occurrence of condensation and evaporation of liquid nitrogen in the mesopores [35]. It is mainly attributed to the difference in mechanism between condensation and evaporation steps found in pores with narrow necks versus wider bodies called ink-bottle pores [36]. The absorption of nitrogen increases linearly until the relative pressure, 0.6, and increases abruptly and then saturates at higher pressure. The capillary condensation occurs at lower relative pressure (P/P_0_ < 0.9), indicating small pore diameter of the microparticles. These pores are mainly present within the primary RF particles [37]. The BET results are summarized in Table 2.

**Figure 4:**
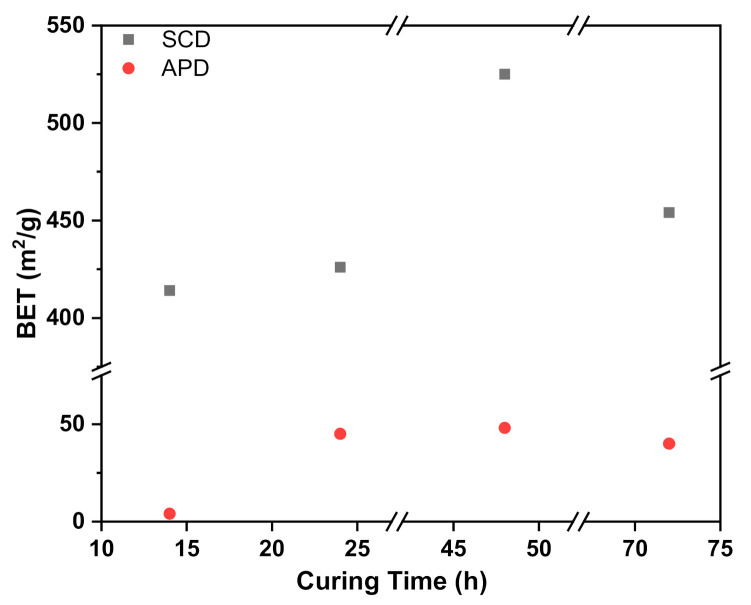
Comparison of BET surface areas of RF microparticles prepared with different curing time under SCD and APD.

Figure 4 depicts the effect of the drying method on BET surface area value of the prepared RF microparticles with different curing times. The ambient pressure dried samples exhibit very low surface area compared to their supercritical dried counterpart. The surface area and total pore volume of the microparticles prepared at 14 h of curing time (S1-SCD) were calculated as 414 m^2^/g and 1.32 cm^3^/g, respectively. Further increase of curing time to 24 h did not alter the surface area and pore volume much. Whereas, increasing the curing time to 48 h (S3-SCD) greatly increased the surface area and pore volume to 525 m^2^/g and 1.74 cm^3^/g, respectively. However, the surface area and pore volume were slightly decreased to 454 m^2^/g and 1.56 cm^3^/g for the RF microparticles cured for 72 h (S4-SCD).

**Figure 5:**
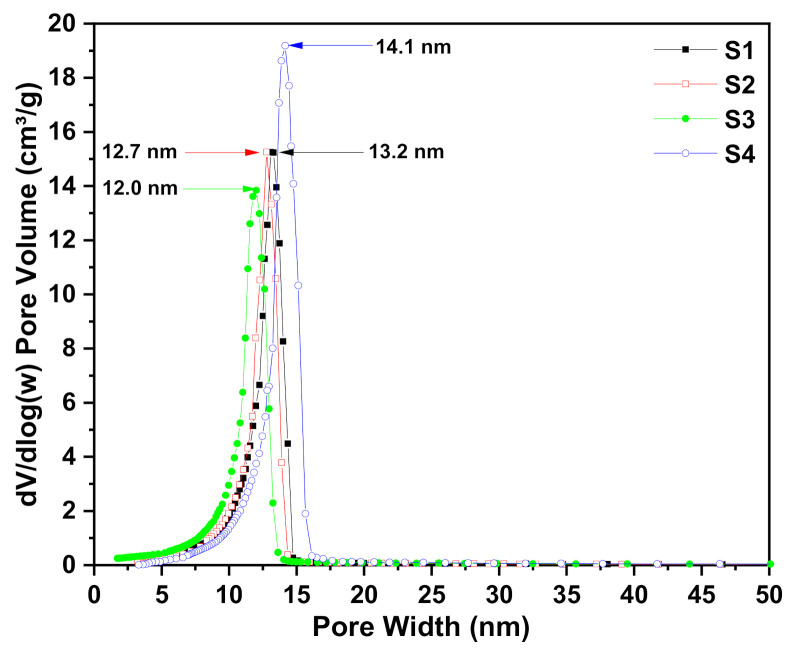
Mesopore size distribution of RF microparticles dried by SCD obtained using BJH model for the N_2_ physisorption isotherms in Figure 3.

The BJH pore size distribution of the RF aerogel microparticles is provided in Figure 5. A narrow pore size distribution was obtained for all the samples. PSD of RF microparticles cured for 14 and 24 h showed a sharp and narrow peak centered at 13.2 and 12.7 nm, respectively. The pore size of the microparticles cured for 48 h slightly decreased to 12.0 nm whereas pore size increased to 14.1 nm while increasing the curing time to 72 h.

The RF microparticles were dried by ambient pressure conditions, resulting in a lower surface area, which can be seen in Figure 4. The ambient pressure drying process of the microparticles greatly affected the gel network and collapsed the pore structure. No pore size distribution was obtained from the physisorption measurements for the abovementioned samples. A detailed comparison of the present RF aerogel microparticles with various reported aerogel microparticles is provided in Table S1 (Supplementary Material).

### 3.4. Morphology of the RF Aerogel Microparticles

**Figure 6:**
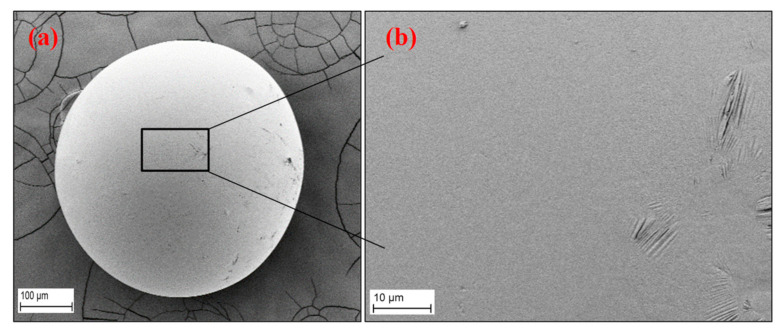
SEM images of a single aerogel microparticle (S2-SCD) under two different magnifications: (**a**) 350× and (**b**) 3000×.

**Figure 7:**
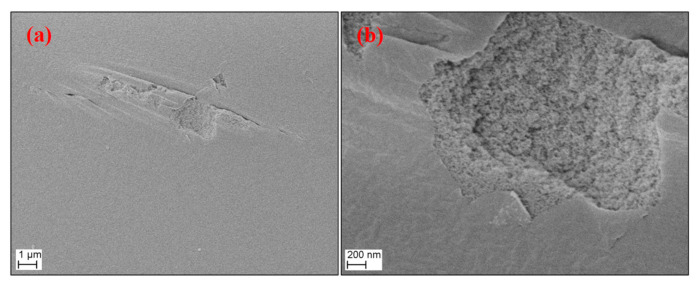
SEM images of the RF microparticles (S4-SCD): (**a**) skin layer on the surface and (**b**) internal structure through a tear in the skin layer.

**Figure 8:**
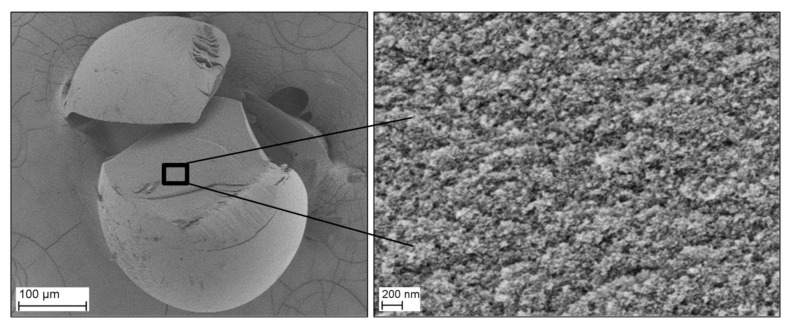
SEM images of internal structure of the microparticle through cross-section (S2-SCD).

**Figure 9:**
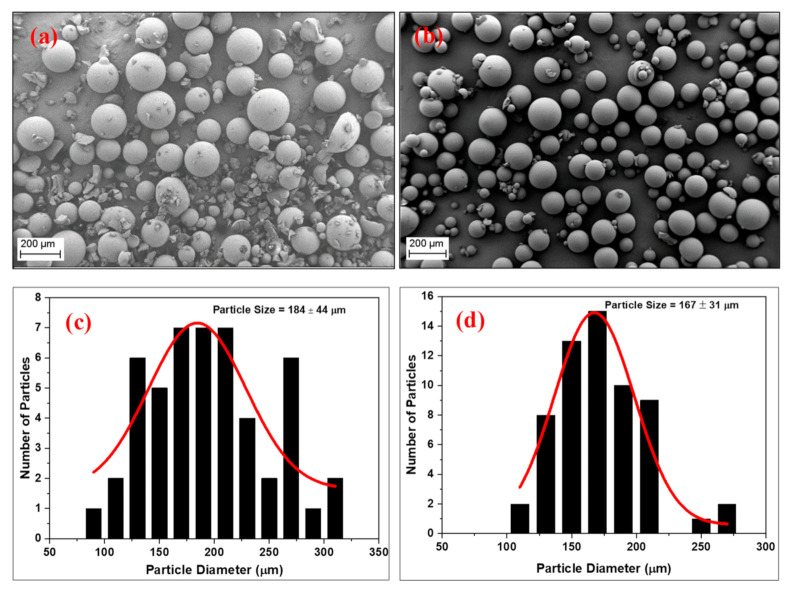
SEM images of the RF aerogel microparticles: (**a**) S2-SCD and (**b**) S2-APD, and their respective particle size distribution curves (**c**,**d**).

The representative SEM images of the RF aerogel microparticles are presented in Figure 6, Figure 7, Figure 8 and Figure 9. SEM image of a single aerogel microparticle has a perfect spherical shape, with a discrete and smooth surface (Figure 6a). Figure 6b shows the close up view of the surface of the microparticles.

RF aerogel microparticles have distinct skin layer morphology on the surface with much denser structures (Figure 7a). The thin skin layer forms at the interface between oil and aqueous RF sol phases due to the fast cross-linking reaction. The interior shows different morphology than the skin layer. The microparticles have denser particle structure below the skin (Figure 7b).

The cross-sectional view of the RF microparticles exhibits porous structure formed by the interconnection of primary nanoparticles (Figure 8). The primary particles are more compact and their interconnection is apparently stronger. The aggregated and combined nature of the primary particle is mainly due to the smaller volume of the droplets formed during the emulsion process. According to Ratke et al. [38], the aggregation and compaction of the particles were in correlation with the drop volume of the RF aerogel droplet gelled in paraffin oil.

RF microparticles have an average size of 128 ± 49 µm (Figure S2, Supplementary Material) for the curing time of 14 h and stirring rate of 200 rpm (S1-SCD). The RF microparticles prepared with stirring rate of 200 rpm, 24 h curing time and RF sol:oil ratio of 1:3 have an average particle diameter of 184 ± 44 µm and 167 ± 31 µm for SCD and APD, respectively (Figure 9). The ambient pressure dried microparticles exhibit diameter shrinkage of approximately 10% only. However, their N_2_ adsorption properties were greatly affected by the drying method, which can be seen in Table 2.

The average particle size of the RF microparticles cured for 48 h (200 rpm, 1:3 ratio) was calculated as 91 ± 45 µm (Figure S3, Supplementary Material). In comparison to RF particles cured for 24 h on the same conditions, the distribution of particles in the range of 100 µm was quite high for S3-SCD. As seen from Table 2, they have very high surface area of 525 m^2^/g and pore volume of 1.74 cm^3^/g among the other prepared RF microparticles. In the case of RF microparticles cured for 72 h, particles were breaking down into smaller sized pieces (Figure S4, Supplementary Material). This is due to the brittle nature of the RF particles upon longer curing time and the impact of the washing step that was employed under stirred conditions before drying. The brittle nature of the RF aerogels was quite common for the Pekala type prepared at a pH of 7 (R/C:200) [39].

### 3.5. Effect of Process Conditions

#### 3.5.1. Stirring Rate

**Figure 10:**
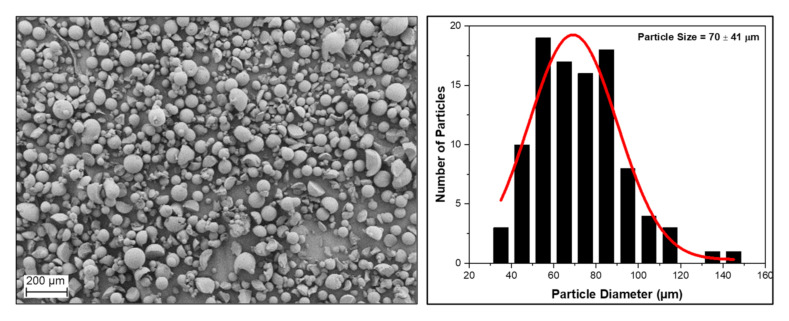
SEM image of the RF aerogel microparticles (S5-SCD) prepared at 500 rpm and its respective size distribution.

The effect of stirring rate was investigated using two different stirring speeds, 200 and 500 rpm to emulsify the system with RF sol:oil ratio of 1:3 and curing time of 24 h. The SEM image and size distribution of the RF microparticles as a function of high stirring rate (500 rpm) are presented in Figure 10. The average particle diameter decreased from 184 ± 44 to 70 ± 41 µm when the stirring speed was increased from 200 to 500 rpm, respectively. This is mainly due to the high energy stirring of the RF sol and oil mixture. The high energy stirring leads to the formation of smaller droplets and also breakage of the bigger droplets into multiple smaller droplets. It is confirmed that the stirring speed has a great influence on the particle size distribution of the formed microparticles. However, their N_2_ adsorption characteristics were not influenced significantly by the stirring speed. The surface area and pore volume were calculated as 429 m^2^/g and 1.22 cm^3^/g with a pore size maxima of 10.2 nm for supercritically dried particles (S5-SCD; Figures S5 and S6, Supplementary). Whereas ambient pressure dried (S5-APD) particles exhibit a very low surface area of 15 m^2^/g with complete loss of the pore structure.

#### 3.5.2. RF Sol:Oil Ratio

**Figure 11:**
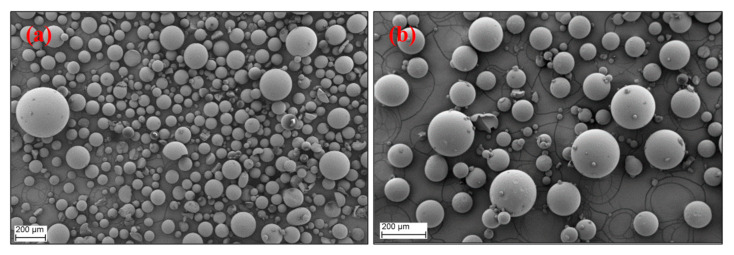
SEM images of RF microparticles prepared with different RF sol:oil ratios: (**a**) S7-SCD (1:1) and (**b**) S6-SCD (1:2).

The effect of RF sol:oil ratio was studied by using three different ratios such as 1:1, 1:2 and 1:3 for preparing different microparticles at 200 rpm over 24 h of curing. The increase of oil phase ratio has a great influence on the particle size distribution of the RF microparticles. As can be seen from the SEM image (Figure 11a), RF sol:oil ratio of 1:1 results in a large number of particles in the range of 75–125 µm with fewer bigger particles of the size of approximately 425 µm. In the case of RF sol:oil ratio of 1:2, particles in the size range of 110–150 µm contribute to the overall size distribution (Figure 11b). As the RF sol:oil phase ratio is increased to 1:3, more particles in the size range of 170–210 µm are produced, leading to an increased average particle size of 184 ± 44 µm. It is evidently seen that the increase in an oil phase increases the particle size. However, their adsorption properties do not depend on the RF sol:oil ratio (Table 3). The sol:oil ratio of 1:2 resulted in microparticles with higher surface area of 543 m^2^/g and pore volume of 1.75 cm^3^/g with supercritical drying (Figure S7, Supplementary Material). Interestingly, the pore size maxima values showed a rising trend with regard to increasing oil phase. The pore size maxima for RF sol:oil ratios of 1:1, 1:2 and 1:3 were found to be 9.9, 11.4 and 12.7 nm, respectively (Figure S8, Supplementary Material).

#### 3.5.3. Initial pH of the RF Sol

**Figure 12:**
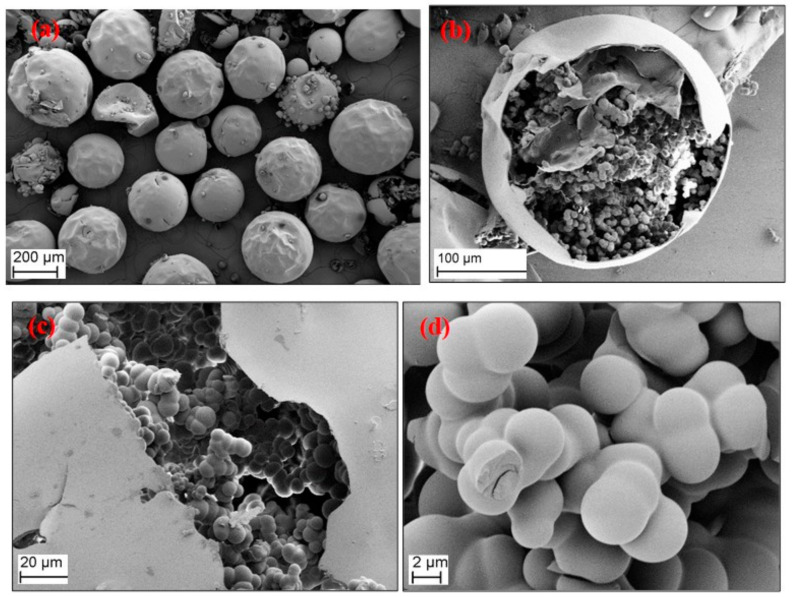
SEM images of RF microparticles prepared with pH adjustment of the sol (S8a-SCD): (**a**) external view of the microparticles (80×) and (**b**–**d**) internal view of the microparticles (**b**) 450×, (**c**) 1K× and (**d**) 5K×.

The initial pH of the RF sol was adjusted from 7 to 5.5 with the addition of 2M HNO_3_ to understand the microstructural and textural properties of the microparticles formed. RF microparticles were obtained only for sol:oil ratio of 1:3 whereas other ratios such as 1:2 and 1:1 did not result in stable microparticle formation; instead, solid precipitate was formed. The size distribution of the microparticles was quite higher (400–700 µm, Figure 12a) compared to microparticles produced without pH adjustment on the same conditions (1:3; 200 rpm; 24 h). The inner cross-section of the microparticles shows an entirely different microstructure (Figure 12b–d). The outer layer was covered with a thick skin shell (approximately 2.5–4.5 µm) and inside was composed of bigger primary particles. The addition of acid to the short base catalyzed step resulted in the form of layer covering the three-dimensional network. The primary particles did not have clear boundaries and grew together forming necks between them. This is in quite good agreement with the primary particle morphology reported for RF aerogels from base-acid catalyzed route by Laskowski et al. [40]. The short base catalyzed step creates small primary particles and a large number of monomers (resorcinol and hydroxy-methyl resorcinol derivative). The small particles are covered by the monomers upon acid addition and further condensation leads to bigger primary particles [40]. The early addition of acid to the base catalyzed RF sol leads to the formation of bigger spherical primary particles of the size of approximately 8 µm (Figure 12d). These bigger primary particles constitute the inner structure of RF microparticles formed in the emulsion process.

RF microparticles prepared with pH adjustment of the sol dried by SCD and APD do not exhibit any characteristic isotherm. The surface area was found to be only 24 and 15 m^2^/g for SCD and APD, respectively. The lower surface area for pH adjusted RF microparticles is in good agreement with the values reported for RF monolithic aerogels catalyzed by base-acid synthesis route [40].

## 4. Conclusions

In conclusion, we prepared RF aerogel microparticles with different sizes, using an emulsion-gelation method. The effects of curing time, stirring rate, sol:oil ratio and initial pH of the sol were investigated on the microparticle formation and its structural and adsorption properties. It was found that increasing stirring rate from 200 to 500 rpm decreased the particle size from 184 ± 44 to 70 ± 41 µm, respectively. The change in initial pH of the sol from 7 to 5.5 altered the inner morphology of the microparticles with bigger primary particles of approximately 8 µm. The higher curing time of 48 h resulted in high surface area and pore volume of 525 m^2^/g and 1.74 cm^3^/g, respectively. The increase in the amount of oil phase to RF sol phase increased the sizes of the microparticles produced. The formation of the polycondensed state of RF was evidenced from the FT-IR spectra. WAXS pattern confirmed the amorphous nature of the RF microparticles formed. The average diameter of the RF microparticles was controlled within a size range of 170–210 µm with a stirring speed of 200 rpm, RF sol:oil ratio of 1:3 and curing time of 24 h. Overall, the size, morphological and adsorption properties of the RF microparticles were tuned by changing the different process conditions as stated above.

## Figures and Tables

**Table 1:** Sample details and experimental conditions.

Sample Codes	R/C	R/F	R/W	Sol:Oil Ratio	Stirring Rate (rpm)	Curing Time (h)	Drying Method *
S1	200	0.74	0.044	1:3	200	14	SCD
							APD
S2						24	SCD
							APD
S3						48	SCD
							APD
S4						72	SCD
							APD
S5				1:3	500	24	SCD
							APD
S6				1:2	200	24	SCD
							APD
S7				1:1			SCD
							APD
S8a *				1:3			SCD
							APD

* SCD = supercritical drying; APD = ambient pressure drying; a = pH adjusted from 7 to 5.5.

**Table 2:** N_2_ physisorption data of the RF microparticles.

Sample Codes	Drying method	S_BET_ (m^2^/g)	V_p_ (cm^3^/g)	D_p_ (nm)
S1	SCD	414	1.32	13.2
	APD	4	0.01	-
S2	SCD	426	1.31	12.7
	APD	45	0.05	-
S3	SCD	525	1.74	12.0
	APD	48	0.14	-
S4	SCD	454	1.56	14.1
	APD	40	0.05	-

(S_BET_ = BET surface area; V_p_ = total pore volume; D_p_ = pore size maxima).

**Table 3:** N_2_ physisorption data of the RF microparticles prepared with different RF sol:oil ratio.

Sample Codes	Sol:Oil Ratio	Drying Method	S_BET_ (m^2^/g)	V_p_ (cm^3^/g)	D_p_ (nm)
S7	1:1	SCD	483	1.39	9.9
		APD	27	0.03	-
S6	1:2	SCD	543	1.75	11.4
		APD	134	0.16	-
S2	1:3	SCD	426	1.31	12.7
		APD	45	0.05	-

## Data Availability

Data sharing not applicable.

## Supplementary Materials

The following are available online at https://www.mdpi.com/article/10.3390/polym13152409/s1, Figure S1: Pictures of the RF microparticles produced by emulsion-gelation method using (a) SCD and (b) APD, respectively, Figure S2: SEM image and its particle size distribution of S1-SCD, cured for 14 h, Figure S3: SEM image and its particle size distribution of S3-SCD, cured for 48 h, Figure S4: SEM images of RF microparticles cured for 72 h (S4-SCD), Figure S5: N_2_ physisorption isotherm of RF microparticles (S5-SCD) prepared at 500 rpm, Figure S6: BJH pore size distribution of RF microparticles (S5-SCD) prepared at 500 rpm, Figure S7: N_2_ physisorption isotherms (77K) of the RF microparticles prepared with RF sol: oil ratios of 1:2 and 1:1, Figure S8: BJH pore size distribution of RF microparticles prepared with RF sol: oil ratios of 1:2 and 1:1; Table S1: Comparisons of the previously reported aerogel microparticles with present RF aerogel microparticles.
