# Supplementary material for: Effect of Process Conditions on the Properties of Resorcinol-Formaldehyde Aerogel Microparticles Produced via Emulsion-Gelation Method
Source: Polymers (Basel). 2021 Jul 22;13(15):2409. doi: 10.3390/polym13152409 (PMC8348565; doi:10.3390/polym13152409)
Supplement: Supplementary file 1 [file polymers-13-02409-s001.zip › polymers-1289091 supplementary final.pdf]

polymers

MDPI

Supplementary Material: Effect of Process Conditions on the Properties of Resorcinol-Formaldehyde Aerogel Microparticles Produced via Emulsion-Gelation Method

Seeni Meera Kamal Mohamed, Charlotte Heinrich, Barbara Milow

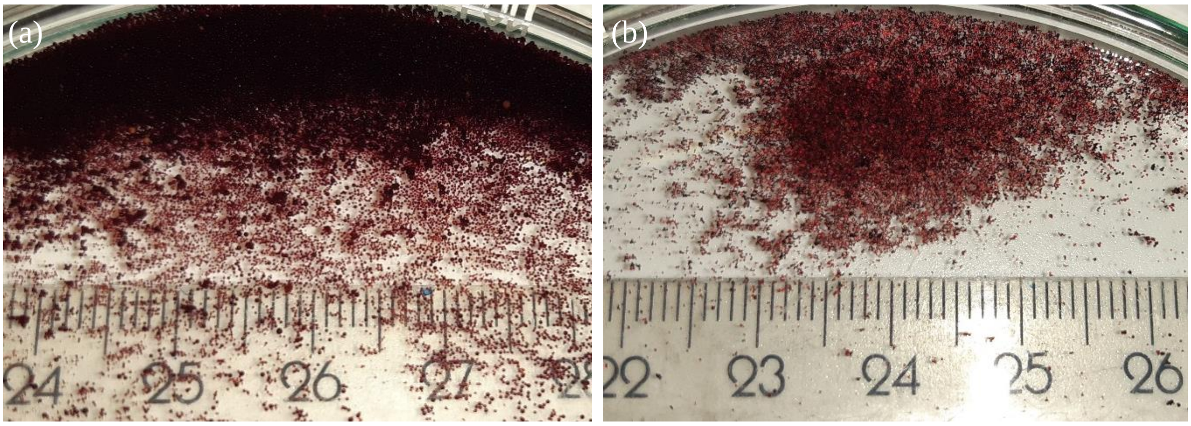

Figure S1. Pictures of the RF microparticles produced by emulsion-gelation method using (**a**) SCD and (**b**) APD, respectively.

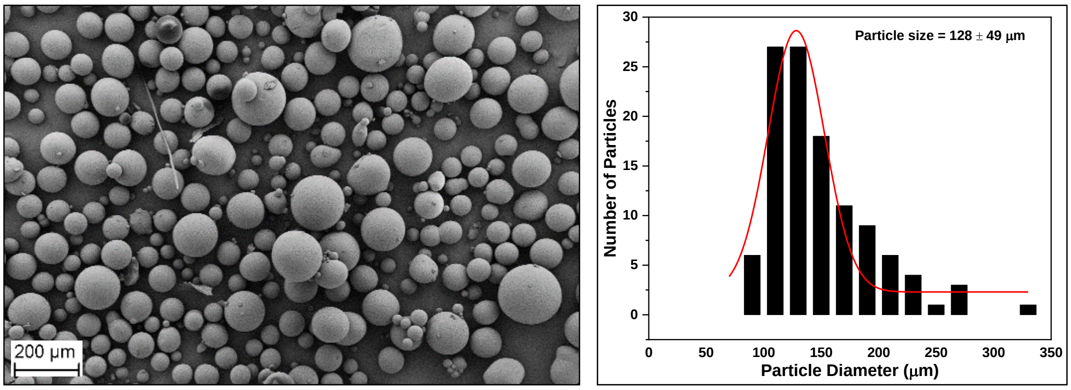

Figure S2. SEM image and its particle size distribution of S1-SCD, cured for 14 h.

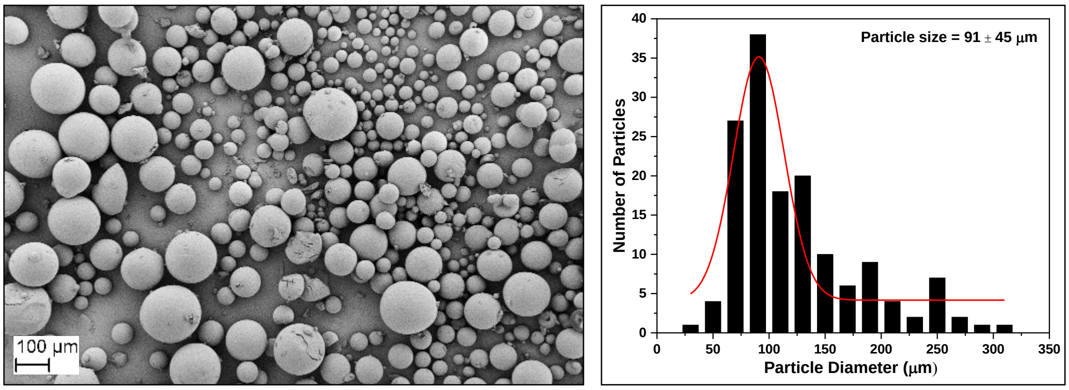

Figure S3. SEM image and its particle size distribution of S3-SCD, cured for 48 h.

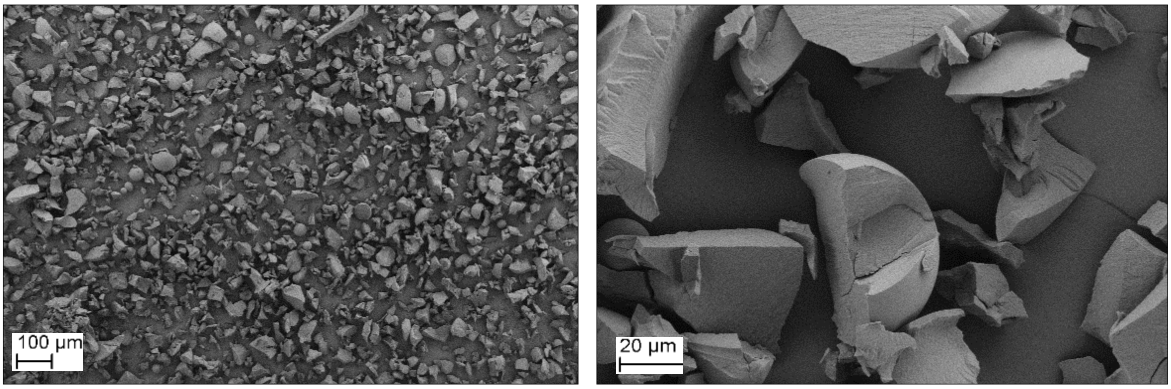

Figure S4. SEM images of RF microparticles cured for 72 h (S4-SCD).

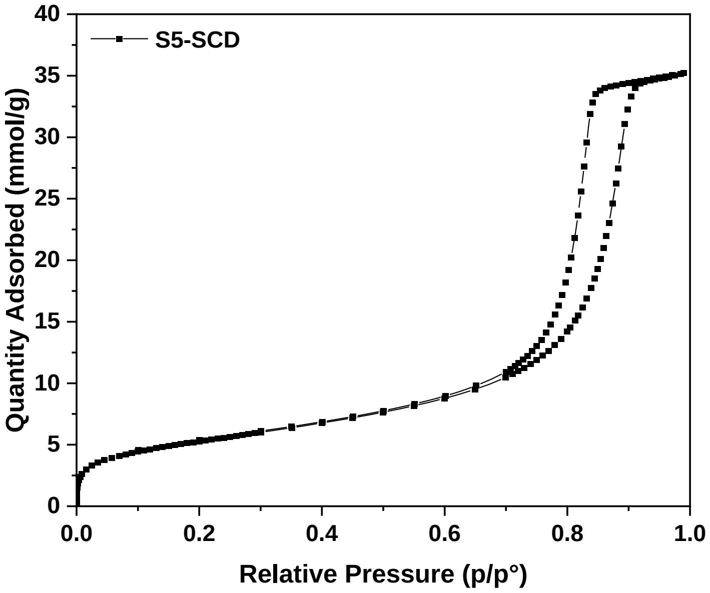

Figure S5. N_2 physisorption isotherm of RF microparticles (S5-SCD) prepared at 500 rpm.

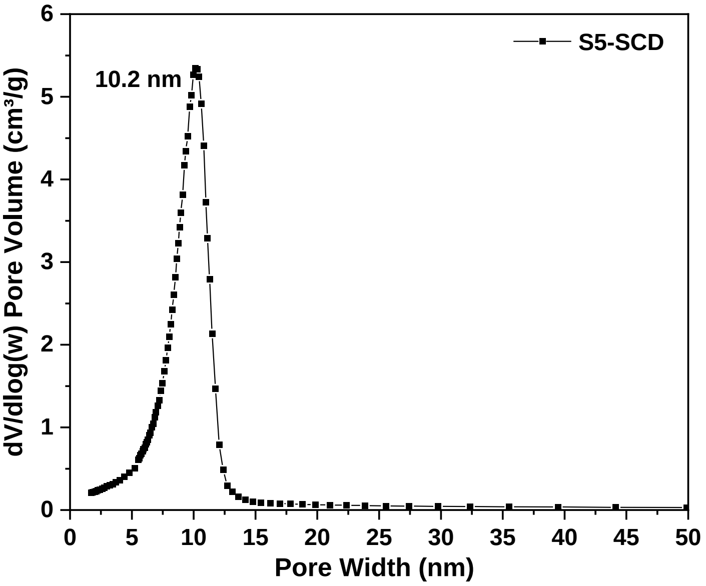

Figure S6. BJH pore size distribution of RF microparticles (S5-SCD) prepared at 500 rpm.

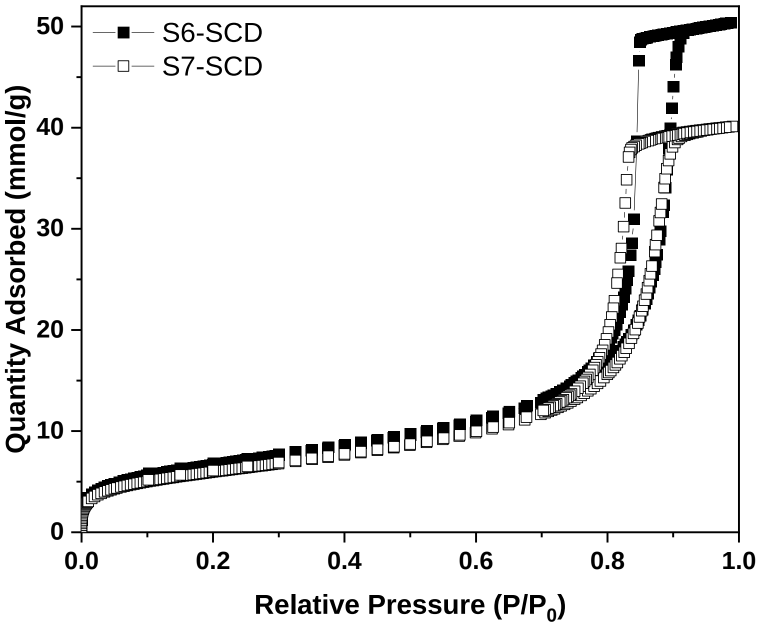

Figure S7. N_2 physisorption isotherms (77K) of the RF microparticles prepared with RF sol: oil ratios of 1:2 and 1:1.

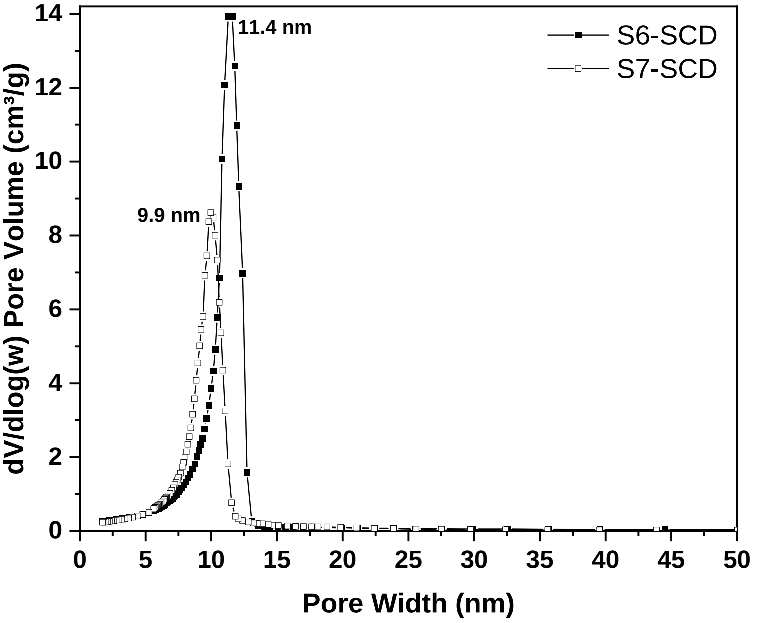

Figure S8. BJH Pore size distribution of RF microparticles prepared with RF sol: oil ratios of 1:2 and 1:1.

Table S1. Comparisons of the previously reported aerogel microparticles with present RF aerogel microparticles.

Sample Type	Oil phase	Size distribution (μm)	Surface area (m^2/g)	Pore volume (cm^3/g)	Avg. Pore diameter (nm)
Alginate [1]	Paraffin oil	7.2-23	330-548	1.7-5.9	-
Cellulose [2]	Paraffin oil	5-20	250-350	-	-
Silica [3]	Edible oil	5	796	-	9.2
Starch [4]	Rapeseed oil	25-270	-	-	-
Starch [5]	Vegetable oil	215-1226	34-120	0.18-0.32	

Polyimide[6]	Silicone oil	200-1000	360-484	-	-
Silica [7]	Hexane	300	853	2.15	16
RF [8]	Cyclohexane	10-80	-	-	-
RF [9]	Paraffin oil	77-158	16-78	-	-
MF* [10]	Dodecane	20-200	200	0.12-0.27	4-12
Carbon (from RF) [11]	Peanut oil	30-1000	250-650	0.1 to 0.4	3.1 to 4.7
Carbon (from RF) [12]	Hexamethylene	2-50	414-603	0.028-0.432	1.43
RF#	Rapeseed oil	50-425	400-526	1.3-1.75	10-14

*Melamine-Formaldehyde; #Current work.

References:

1. Gonçalves, V.S.S.; Gurikov, P.; Poejo, J.; Matias, A.A.; Heinrich, S.; Duarte, C.M.M.; Smirnova, I. Alginate-based hybrid aerogel microparticles for mucosal drug delivery. *European Journal of Pharmaceutics and Biopharmaceutics* **2016**, *107*, 160-170, doi:https://doi.org/10.1016/j.ejpb.2016.07.003.
2. Druel, L.; Kenkel, A.; Baudron, V.; Buwalda, S.; Budtova, T. Cellulose Aerogel Microparticles via Emulsion-Coagulation Technique. *Biomacromolecules* **2020**, *21*, 1824-1831, doi:10.1021/acs.biomac.9b01725.
3. Gong, Y.; Lei, Y.; Chen, X.; Song, H.; Liu, R. A novel silica aerogel microspheres loaded with ammonium persulfate gel breaker for mid-deep reservoirs. *Journal of Sol-Gel Science and Technology* **2018**, *88*, 105-113, doi:10.1007/s10971-018-4787-7.
4. Baudron, V.; Taboada, M.; Gurikov, P.; Smirnova, I.; Whitehouse, S. Production of starch aerogel in form of monoliths and microparticles. *Colloid and Polymer Science* **2020**, *298*, 477-494, doi:10.1007/s00396-020-04616-5.
5. García-González, C.A.; Uy, J.J.; Alnaief, M.; Smirnova, I. Preparation of tailor-made starch-based aerogel microspheres by the emulsion-gelation method. *Carbohydrate Polymers* **2012**, *88*, 1378-1386, doi:https://doi.org/10.1016/j.carbpol.2012.02.023.
6. Teo, N.; Jana, S.C. Surfactant-Free Process for the Fabrication of Polyimide Aerogel Microparticles. *Langmuir* **2019**, *35*, 2303-2312, doi:10.1021/acs.langmuir.8b03841.
7. Yun, S.; Luo, H.; Gao, Y. Superhydrophobic silica aerogel microspheres from methyltrimethoxysilane: rapid synthesis via ambient pressure drying and excellent absorption properties. *RSC Advances* **2014**, *4*, 4535-4542, doi:10.1039/C3RA46911E.
8. Alviso, C.T.; Pekala, R.W.; Gross, J.; Lu, X.; Caps, R.; Fricke, J. Resorcinol-Formaldehyde and Carbon Aerogel Microspheres. *MRS Proceedings* **1996**, *431*, 521, doi:10.1557/PROC-431-521.
9. Mulani, K.; Patil, V.; Chavan, N.; Donde, K. Adsorptive removal of chromium(VI) using spherical resorcinol-formaldehyde beads prepared by inverse suspension polymerization. *Journal of Polymer Research* **2019**, *26*, 41, doi:10.1007/s10965-019-1705-9.
10. Schwarz, D.; Weber, J. Synthesis of mesoporous poly(melamine-formaldehyde) particles by inverse emulsion polymerization. *Journal of Colloid and Interface Science* **2017**, *498*, 335-342, doi:https://doi.org/10.1016/j.jcis.2017.03.064.
11. Liu, N.; Zhang, S.; Fu, R.; Dresselhaus, M.S.; Dresselhaus, G. Carbon aerogel spheres prepared via alcohol supercritical drying. *Carbon* **2006**, *44*, 2430-2436, doi:https://doi.org/10.1016/j.carbon.2006.04.032.
12. Wang, X.; Wang, X.; Liu, L.; Bai, L.; An, H.; Zheng, L.; Yi, L. Preparation and characterization of carbon aerogel microspheres by an inverse emulsion polymerization. *Journal of Non-Crystalline Solids* **2011**, *357*, 793-797, doi:https://doi.org/10.1016/j.jnoncrysol.2010.11.015.
